# Physical and Fitness Characteristics of Elite Professional Rugby Union Players

**DOI:** 10.3390/sports8060085

**Published:** 2020-06-05

**Authors:** Logan Posthumus, Campbell Macgregor, Paul Winwood, Katrina Darry, Matthew Driller, Nicholas Gill

**Affiliations:** 1 Faculty of Health, Education and Environment, Toi Ohomai Institute of Technology, Tauranga 3112, New Zealand; campbell.macgregor@toiohomai.ac.nz (C.M.); paul.winwood@toiohomai.ac.nz (P.W.); 2 Te Huataki Waiora School of Health, The University of Waikato, Hamilton 3216, New Zealand; nicholas.gill@nzrugby.co.nz; 3 New Zealand Rugby Union, Wellington 6011, New Zealand; katrina.darry@nzrugby.co.nz; 4 School of Applied and Social Sciences, Central Queensland University, Rockhampton 4701, Australia; 5 School of Sport and Recreation, Auckland University of Technology, Auckland 0627, New Zealand; 6 School of Allied Health, Human Services and Sport, La Trobe University, Melbourne 3086, Australia; m.driller@latrobe.edu.au

**Keywords:** anthropometrics, body composition, strength, speed, power, aerobic fitness

## Abstract

This study explored the physical and fitness characteristics of elite professional rugby union players and examined the relationships between these characteristics within forwards and backs. Thirty-nine elite professional rugby union players from the New Zealand Super Rugby Championship participated in this study. Body composition was measured using dual-energy X-ray absorptiometry alongside anthropometrics. Fitness characteristics included various strength, power, speed, and aerobic fitness measures. Forwards were significantly (*p* ≤ 0.01) taller and heavier than backs, and possessed greater lean mass, fat mass, fat percentage, bone mass, and skinfolds. Forwards demonstrated greater strength and absolute power measures than backs (*p* = 0.02), but were slower and possessed less aerobic fitness (*p* ≤ 0.01). Skinfolds demonstrated very *large* correlations with relative power (*r* = −0.84) and speed (*r* = 0.75) measures within forwards, while backs demonstrated *large* correlations between skinfolds and aerobic fitness (*r* = −0.54). Fat mass and fat percentage demonstrated *very large* correlations with speed (*r* = 0.71) and aerobic fitness (*r* = −0.70) measures within forwards. Skinfolds, fat mass, and fat percentage relate strongly to key fitness characteristics required for elite professional rugby union performance. Individual and positional monitoring is important due to the clear differences between positions.

## 1. Introduction

Rugby union (RU) is a high contact field-based team sport which consists of an 80-min game consisting of two 40-min halves separated by a break no longer than 15 min. Each team has 15 players on the field at a time, comprising eight forwards (numbers 1–8) and seven backs (numbers 9–15). The team also has eight reserves on the side line (bench) who can be substituted only once during the game [1]. The game itself is composed of collisions and intermittent exercise, where short periods of maximal or high intensity activity (e.g., sprinting, tackling, rucking, scrummaging, mauling) last between 5 and 15 s, interspersed with activity of lower intensities (e.g., standing, walking, jogging) of up to 40 s [2]. During a game, players will generally cover approximately 6500 to 7500 m, with an average heart rate around 172 bpm [3,4].

The sport of RU requires a range of fitness characteristics, such as strength, power, speed, and aerobic fitness, in order to perform well and meet the demands of the sport [5,6]. Players all possess varying levels of physical characteristics, such as anthropometric (height, body mass, skinfold) and body composition (lean mass, fat mass, bone mass) in order to achieve adequate levels of the desired aforementioned fitness qualities and to meet specific positional demands of the sport [7,8,9]. Numerous studies have shown that these fitness [5,7,10] and physical characteristics [11,12,13,14,15,16] vary greatly depending on the player’s position within the team. In general, forwards have a greater body mass and strength emphasis in order to scrummage with more force, tolerate more collisions and gain and retain possession of the ball [11]. Meanwhile, backs generally have less body mass, a more efficient lean to fat mass ratio, and more emphasis on speed and manoeuvrability in order to utilise possession of the ball and create scoring opportunities from more open spaces [1].

The importance of anthropometrics on team success in professional RU has been investigated [17,18]. The consensus from these studies suggests that having larger players, particularly heavier forwards and taller backs, generally leads to greater team success in international World Cup competitions. Fitness and physical characteristics have also been explored in relation to game statistics deemed important for success [6] in which body composition, speed, repeated sprint ability, and strength are important factors in the performance of key game behaviours during competition. In particular, Smart et al. [6] suggested that decreasing percent body fat may improve work rate and the ability to repeatedly perform tasks in competition, especially the important aforementioned fitness qualities listed within our study. Therefore, it is no surprise that as playing level increases from semi-professional to professional, players are heavier, have greater lean mass, and lower skinfold and percent body fat scores, while demonstrating greater strength, power and speed [10].

Although a range of studies have reported the physical [11,12,13,14,15,16] and fitness [5,7,10] characteristics of professional RU players and have suggested that body composition with optimal amounts of lean mass and fat mass are important for specific fitness qualities, there is limited literature directly examining the relationships between physical and fitness characteristics within professional RU forwards and backs. Furthermore, physical characteristics in the form of body composition have traditionally been collected using skinfold measurements, which are a practical measure due to their reliability, ease of use, low cost, and portability [13,19]. However, a method that has gained popularity for body composition analysis within professional athletes [20,21] and specifically within professional rugby players [11,12,13,14,15,16,19,22,23] is the use of dual-energy X-ray absorptiometry (DXA).

Gaining a greater understanding of the relationships between physical and fitness characteristics is important to better comprehend the athletic profile of these elite professional RU players. This may allow for more detailed nutritional and physical training programmes not only for current professional players, but for future RU players progressing through academies and development programmes in order to optimise important physical and fitness characteristics. Therefore, the aim of this study was to; (a) explore the physical and fitness characteristics of elite professional RU players and (b) investigate the relationship between physical and fitness characteristics within forwards and backs.

## 2. Materials and Methods

### 2.1. Participants and Study Design

Thirty-nine elite male professional RU players (26.6 ± 3.3 y, 187.6 ± 7.6 cm, 108.0 ± 14.1 kg) playing in the NZ Super Rugby Championship were recruited for this study. Professional experience for players was 7.3 ± 2.9 y, which was quantified by determining when players were first selected for a professional rugby team (e.g., Province/Franchise). Players were categorised into forwards and backs for analysis and comparison. Forwards were comprised of; hookers (*n* = 4), props (*n* = 6), locks (*n* = 5), and loose forwards (*n* = 8). Backs were comprised of; half-backs (*n* = 3), first-fives (*n* = 3), midfield (*n* = 5), and outside backs (*n* = 5).

Data were collected using a cross-sectional study design, in which all testing took place at the end of the Super Rugby Championship pre-season. All anthropometric, body composition and fitness testing took place in the same week, with DXA and surface anthropometry measures being collected on the same day. All participants provided informed consent and the research was approved by the University of Waikato Human Research Ethics Committee (2019#02).

### 2.2. Anthropometrics

Upon waking and following a voided bladder, participants were then asked to change into only their underwear or tight-fitting sport shorts. Body mass measurement was obtained using a set of portable electronic scales (SECA, Birmingham, UK) to 0.1 kg accuracy. Thereafter, height was assessed using a portable stadiometer (SECA, Birmingham, UK) to 0.5 cm accuracy. The sum of eight site skinfold measurements were then carried out by the same Level 1 International Society for the Advancement of Kinanthropometry (ISAK) accredited anthropometrist, using Harpenden callipers (British Indicators, Hertfordshire, UK) to 0.1 mm accuracy. Skinfold measurements were made on the right side of the body using ISAK techniques previously described [24], with a sum of eight skinfolds calculated from the measures of the; biceps, triceps, subscapular, abdominal, supraspinale, iliac crest, mid-thigh and medial calf skinfold sites. Duplicate measures were carried out for all sites to determine re-test reliability, in which a third measure was recorded if the difference between duplicate measures was greater than 4% for a single skinfold [11]. Anthropometrical technical errors of measurement were below the recommended limits [25], and all anthropometric equipment was calibrated as per the manufacturers’ guidelines.

### 2.3. Body Composition

Total body composition (lean mass, fat mass, fat percentage, bone mass and bone mineral density) was measured using a fan-beam DXA scanner (Hologic Discovery A, Hologic, Bedford, MA, USA), with analysis performed using Apex 4.6.0.3 software (Hologic, Bedford, MA, USA). All scanning procedures were standardised for all participants following the guidelines of the DXA manufacturer and the standards outlined by the International Society for Clinical Densitometry. The same qualified technician performed all measurements for positioning consistency. The scanner was tested for consistent calibration daily as per the manufacturer’s guidelines for quality control purposes. All scans were undertaken using the array mode and all participants were scanned on the same day within a five-hour window (0830–1330 h), with all participants consuming breakfast and fluids at the same time (0700 h).

All protocols followed previously described techniques to maximise technical reliability and minimise error [21,26], however, participants were unable to be scanned in a fasted state. Participants were required to remove all metal items from their body and were scanned wearing tight-fitting sport shorts or underwear, free from zips, studs and/or metal objects. Participants were then instructed to lay supine on the scanning bed as still as possible for the duration of the scan. The same technician that performed the scan manually segmented the whole-body scan into regions during the analysis process. The head was included in the analysis for all total body measures. Two participants that were too tall for the scanning bed had their feet removed from the scan as advised by Hologic. This allowed the head to be included in the scan, which is recommended to offset the remaining tissue percentages [27].

### 2.4. Fitness Testing

All fitness testing took place on the same day using methods previously described [5,28,29]. Speed and aerobic fitness tests were carried out in the morning, while strength and power tests were performed in the afternoon five hours later. Before performing the sprints followed by aerobic fitness tests in the morning, participants completed a 15-min warm-up following the same procedure as Ross et al. [28]. Prior to performing the power followed by strength tests in the afternoon, participants completed a 20-min warm up which consisted of foam rolling and dynamic functional movements.

#### 2.4.1. Strength

Strength was quantified via one repetition maximum (1RM) assessments in the squat, bench press and weighted chin-up. For the squat, participants selected either a barbell front or back squat and self-selected their stance width and were required to reach “parallel” for the lift to be counted (hip crease level with knee cap). For the bench press, participants used a pronated self-selected grip width on a barbell and were required to lower the bar to chest level with feet and glutes remaining in contact with the floor and bench at all times. Participants commenced the chin up with arms extended in a supinated grip and were required to raise themselves to where their chin was over the bar in order for the lift to be deemed successful. Squat and bench press 1RMs were calculated based on the amount of external resistance lifted only. Meanwhile, chin up 1RM was calculated as bodyweight plus added external weight (Chin Up BW+AW), in which a dip-belt was used to load external resistance. All participants were instructed to warm up with three sets of sub-maximal loads (3–5 repetitions at 50%, 60%, 70% of perceived maximum strength), and thereafter, progressively increase resistance on each exercise until they reached their 1RM, with approximately three minutes rest between attempts [5,29].

#### 2.4.2. Power

Vertical power was collected by having participants perform maximal countermovement jumps (CMJ). The bodyweight condition (CMJPP) was performed while holding a 1-kg pole, while the loaded condition (CMJ40PP) was performed using a 40-kg barbell. A linear position transducer (GymAware, Kinetic Performance Technology, Canberra, Australia) was attached to the pole or bar in order to assess peak power (PP). Participants lowered to a self-selected squat depth and explosively jumped as high as possible while maintaining hold of the pole or barbell. Three practice jumps were performed by the participants, followed by three maximal jumps for CMJ and CMJ40 with three minutes rest between trials. For analysis, the highest value recorded by each participant was included. Relative power (W/kg) was also calculated for the bodyweight condition (CMJRP) and loaded condition (CMJ40RP) by dividing each participant’s PP by their body mass. Horizontal power was indirectly measured using a broad jump with participants following the same protocol as used for vertical power. The broad jump was performed with full arm swing, feet hip width, and toes on a marked line. A tape measure was used to determine jump distance from toe-off to the back of the most rear foot, to the nearest centimetre. In order for the jump to be included in the analysis, in which the furthest jump attempt was used, participants were required to land on two feet without falling.

#### 2.4.3. Speed

Speed was measured outdoors on an artificial (turf) running surface, in which participants performed a 20 m maximal-effort sprint. Dual-beam electronic timing lights (Speedlight TT, Swift Performance Equipment, Lismore, Australia) were set up at 0, 10, and 20 m distances. From a crouched, split-stance position with no countermovement or “rocking’ motion, participants began the sprint 30 cm behind the starting gate. At each split, speed was recorded to the nearest 0.01 s. Two sprints were performed, with five minutes rest between efforts, with the fastest sprint by each participant being used for analysis.

#### 2.4.4. Aerobic Fitness

The 20 m Yo-Yo intermittent recovery 1 (IR1) test was carried out on an artificial (turf) running surface to assess the participants’ aerobic fitness. The IR1 was completed according to previously described methods [30]. Participants were asked to complete two 20 m shuttles before a beep was emitted from a stereo at each end, followed by 10 s of active recovery between shuttles. Over the course of the test, the required speed for each shuttle gradually increased, in which participants were required to run until they were no longer able to complete the shuttles in time with the beeps. When a participant failed twice to reach the designated lines before the beeps, the end of the test was considered. For analysis, the final level and shuttle reached was converted to total distance covered (m) for each participant.

### 2.5. Statistical Methods

All athlete characteristics are expressed as means and standard deviations (mean ± SD). Differences between forwards and backs were compared using an independent *t*-test. Significance was set as *p* < 0.05. Positional differences with 95% confidence limits (95% CL) were calculated for all measures. Effect sizes (ES) were calculated with the following quantitative thresholds: *trivial* < 0.20, *small* 0.21–0.60, *moderate* 0.61–1.20, *large* 1.21–1.99 and *very large* > 2.0 [31]. Correlation coefficient were calculated to determine relationships between physical and fitness characteristics. The magnitude of the correlations were rated as *trivial* (< 0.09), *small* (0.10–0.29), *moderate* (0.30–0.49), *large* (0.50–0.69), *very large* (0.70–0.89), or *nearly perfect* (0.90–0.99) [32]. All statistical analyses were conducted using SPSS v24 for Windows (IBM, New York, NY, USA).

## 3. Results

### 3.1. Physical Characteristic Comparison

The demographics and physical characteristics between forwards and backs can be observed in Table 1. Forwards were significantly (*p* ≤ 0.01) taller (ES = 1.20) and heavier (ES = 2.10) than backs. Forwards possessed significantly (*p* ≤ 0.01) greater lean mass (ES = 2.06), fat mass (ES = 1.87), fat percentage (ES = 1.51), bone mass (ES = 1.45), and skinfolds (ES = 1.51) than backs. Forwards were older, more experienced and possessed greater bone mineral density than backs; however, no significant differences were present.

### 3.2. Fitness Characteristic Comparison

Fitness characteristics between forwards and backs can be observed in Table 2. Forwards demonstrated significantly greater 1RM strength in the squat (*p* = 0.02; ES = 0.81), bench press (*p* = 0.02; ES = 0.81), and chin up (*p* = 0.01; ES = 0.90) compared to backs. Forwards also demonstrated significantly greater CMJPP (*p* ≤ 0.01; ES = 1.15) and CMJ40PP (*p* ≤ 0.01; ES = 0.99) compared to backs. Conversely, forwards possessed lower CMJRP (*p* = 0.25; ES = −0.38) and CMJ40RP (*p* = 0.06; ES = −0.64) compared to backs, although differences were not significant. Forwards demonstrated significantly less broad jump distance (*p* = 0.02; ES = −0.79), slower 10 m sprint (*p* ≤ 0.01; ES = 1.99), and 20 m sprint times (*p* ≤ 0.01; ES = 1.90) and covered less distance in the Yo-Yo test (*p* ≤ 0.01; ES = −2.56) compared to backs.

### 3.3. Characteristic Relationships

The relationships between physical and fitness characteristics can be observed in Table 3. *Trivial* to *very large* correlations existed between physical and fitness characteristics within forwards and backs. Of the 1RM strength measures, the squat demonstrated *small* to *large* correlations with anthropometric (*r* = −0.22–0.59) and body composition (*r* = 0.14–0.69) measurements for forwards. Within backs, 1RM strength measures demonstrated *trivial* to *large* correlations with anthropometrics (*r* = 0.03–0.88) and body composition (*r* = 0.08–0.89) measures, in which chin up demonstrated the strongest relationships with body mass (*r* = 0.88) and lean mass (*r* = 0.89).

In relation to power measures, the strongest correlations observed within forwards and backs were CMJRP (*r* = −0.82) and CMJ40RP (*r* = −0.85). The strongest correlations for forwards were between CMJRP, CMJ40RP and Sum8SF (*r* = −0.82–−0.84, respectively), while backs demonstrated strongest relationships between CMJRP, CMJ40RP and body mass (*r* = −0.70–−0.85, respectively). Regarding speed measures, both 10 and 20 m sprint times shared their largest correlations with Sum8SF (*r* = 0.75) and fat mass (*r* = 0.69–0.71, respectively) within forwards. The largest correlations for backs in relation to 10 and 20 m sprint was fat percentage (*r* = 0.40) and age (*r* = 0.51), respectively. In relation to aerobic fitness, forwards’ Yo-Yo distance correlated most strongly with fat mass (*r* = −0.66) and fat percentage (*r* = −0.70), while backs’ Yo-Yo distance correlated most strongly with Sum8SF (*r* = −0.54).

## 4. Discussion

This study explored the physical and fitness characteristics of elite professional RU players in NZ. To the authors’ knowledge, this is the first study to examine the relationships between these characteristics within elite professional RU players. Within this study, forwards were substantially bigger and stronger in all areas and possessed greater absolute power compared to backs. In contrast, backs were leaner, faster, aerobically fitter, and possessed greater relative power and broad jump distance. When examining the relationships between physical and fitness characteristics, skinfold measures were most consistently correlated with power and speed measures for forwards, showing *large* to *very large* correlations. Skinfolds, fat mass and percent body fat within forwards were most consistently correlated with relative power, speed and Yo-Yo distance, showing *large* to *very large* correlations. Moreover, backs demonstrated *large* to *very large* correlations for body mass, lean mass and fat mass in relation to strength and relative power measures. In addition, *large* correlations were observed between age and 20 m sprint, and between skinfolds and Yo-Yo distance within backs.

These findings support previous studies which demonstrated forwards as having different body composition [11,12,13,15,16] and anthropometric characteristics than backs, including greater strength and absolute power measures, but slower sprint performance and less aerobic fitness [6,7,10,33]. The characteristic differences between forwards and backs can be attributed to specific positional demands, in which being larger and stronger can assist forwards with winning line outs, scrumming with more force [34], gaining and retaining possession of the ball at break downs [6], and absorbing more collisions through tackles and ball carries [4]. Conversely, backs need to be leaner, faster, and aerobically fitter in order to defend or out manoeuvre their opponent in more open spaces, cover more distance, perform more sprints, and ultimately finish phases with trying scoring opportunities [4,6]. However, as the game has evolved, the versatility of backs has increased by performing more additional tasks typically carried out by forwards (e.g., turnovers, clearing rucks) [11]. It has been suggested that teams with larger players (in particular larger forwards and taller backs) have greater success in test matches and World Cup competitions [17,18].

Studies within southern [6,11,12,13] and northern hemisphere [7,15] professional RU players demonstrated similar anthropometric, body composition, and fitness characteristic differences between forwards and backs, as seen in the current study. Interestingly, both forwards and backs within our study demonstrated greater height, body mass, lean mass, and fat mass, but possessed less bone mass and percent body fat compared to professional RU players observed in the northern hemisphere (United Kingdom) [15]. Meanwhile, similar strength and speed measures were observed between the current study and northern hemisphere players [7]. Within the southern hemisphere, similar strength and speed measures were observed in a previous study conducted in NZ [6], while similar heights, lean mass and bone mass were observed between professional NZ and Australian players. However, less fat mass and percent body fat were observed in Australian players [11,12,13]. These findings suggest that anthropometric, body composition, and fitness characteristic differences between forwards and backs are very similar within northern and southern hemisphere teams, though variances may be due to differences in environmental conditions, game-play [35,36], strength and conditioning practices [37], and ethnic/cultural make-up [11,12,13,19].

Increasing body mass, in particular lean mass, while reducing fat mass is a common goal within professional RU players. Excess fat mass may attenuate force production and increase energy expenditure, leading to decreased strength, power, and speed while potentially increasing fatigue [12]. This is supported by the literature examining the relationships between physical fitness and game behaviours, in which work rate and the ability to repeatedly perform tasks may be improved by reducing percent body fat [6]. The results of our study demonstrate that higher skinfold, fat mass and fat percentage correlated strongly with decreased power, speed, and aerobic fitness measures within forwards. These results suggest that leaner forwards may possess more favourable fitness characteristics; however, too much body mass, regardless of composition, may lead to decreased power, slower sprint times, and less distance covered in the Yo-Yo. Nevertheless, certain positions (e.g., props, locks, loose forwards) may require additional fat mass to withstand the forces associated with scrummaging and high frequency collisions [23], though more research is required within this area. Meanwhile, greater body mass and lean mass within backs correlated with greater strength, but lower relative power, which could partly be due to increased overall body mass and fat mass [12,15]. Furthermore, fat mass and fat percentage within backs demonstrated only *small* to *moderate* correlations with speed and aerobic fitness measures compared to *large* and *very large* correlations observed within forwards. Additionally, *large* correlations between skinfolds and aerobic fitness were observed within forwards and backs, suggesting greater skinfolds relate to less Yo-Yo distance.

Interestingly, Smart et al. [6] suggested that greater emphasis may need to be applied to speed and acceleration, especially over 10 m, due to the relationships observed with the number of line breaks, tackle breaks and tries scored, which are important game behaviours for successful phase and team outcomes. Our study observed that forwards with more skinfolds, fat mass and fat percentage may possess slower sprint times; therefore, both fat and skinfold measures appear important individual tracking tools, especially for forwards in order to improve speed measures. Additionally, lower skinfolds, fat mass and fat percentage may also improve aerobic fitness (Yo-Yo distance), which was also a key variable influencing game-play [6]. It is important to note that there is no specific target for anthropometrics, body composition, and fitness characteristics for professional RU players. The physical and fitness aspect of a player is only one component of performance. The ability to read game-play, make the right decisions quickly, and connect with other players are also very important aspects relating to performance [1,6].

Optimising physical and fitness characteristics for the individual may allow players to execute skill and tactical attributes with even greater success [6]. Numerous studies have shown large physical and fitness changes following a pre-season training block [7,12,29], demonstrating increases in lean mass while decreasing fat mass. Although it appears that professional RU players generally maintain body mass throughout the in-season period, in numerous players, lean mass is lost and substituted by an increase in fat mass [15]. Therefore, decreases in fitness characteristics may also occur due to the relationships observed with lean mass, fat mass and skinfolds. However, more research is needed to determine changes in physical and fitness characteristics throughout a season.

Future studies could monitor both physical and fitness characteristics throughout the in-season period to determine what effect changes in body composition may have on fitness characteristics. This would help profile individual players and assist in optimising strength and conditioning and nutrition programmes. Future studies could also explore different methods for maintaining lean mass while reducing increases in fat mass during in-season periods. It is well documented that professional RU players demonstrate great increases in lean mass and decreases in fat mass during pre-season periods [7,12,29] and appear to increase body mass, lean mass index, and strength measures over consecutive seasons [38]. However, players may be able to improve further by reducing losses in lean mass and increases in fat mass during the in-season.

A limitation within the current study was that players were not able to be in an overnight fasted state for DXA scans, which is suggested for best practice [21,26]. Though not ideal, food and fluid intakes remained below 500 g for each player, which has been shown to minimise biological measurement error when using DXA [39]. Nevertheless, we acknowledge some error may be present across the whole group, though all players were under the same conditions. Furthermore, scanning the head and body separately followed by adding the partial scans together is potentially a more accurate method for taller individuals than excluding the feet from the scan, as carried out in two of our players. Although minimal, it is important to note that adding partial scans can increase scan time and exposure to radiation [40]. Lastly, our study was limited in that we could only use a small sample size of elite players and could only group players according to their primary positional group (forwards and backs). A larger sample would have allowed further exploration of forwards (front row, second row, back row) and backs (inside backs, midfield, outside backs) or separate positions within these classifications. This would be useful given the unique differences in roles between each position within a RU team [10].

## 5. Conclusions

In conclusion, we report that there are significant differences in the physical and fitness characteristics between NZ elite professional RU forwards and backs. The present study also reported that strength, power, speed, and aerobic fitness measures were most strongly correlated with skinfolds, fat mass, and fat percentage within both forwards and backs. This information may be useful for practitioners developing future professional RU players, and to help monitor and optimise training and nutritional programmes in current professional RU players.

## Figures and Tables

**Table 1:** Physical characteristics of elite professional rugby union (RU) players.

	All Players (*n* = 39)	Position		% Difference (±95% CL)	Effect Size (±95% CL)	Qualitative Inference
		Forwards (*n* = 23)	Backs (*n* = 16)			
Demographics						
Age (y)	26.6 ± 3.3	27.2 ± 2.8	25.7 ± 3.8	6.0 ± 9.2	0.47 ± 0.33	*Small* +
Experience (y)	7.3 ± 2.9	7.7 ± 2.8	6.7 ± 3.1	15.7 ± 35.4	0.36 ± 0.33	*Small* +
Anthropometrics						
Height (cm)	187.6 ± 7.6	190.9 ± 5.9 *	183.0 ± 7.5	4.3 ± 2.5	1.20 ± 0.35	*Moderate* +
Body Mass (kg)	108.0 ± 14.1	116.5 ± 10.1 *	95.9 ± 9.4	21.5 ± 7.4	2.10 ± 0.40	*Very Large* +
Sum8SF (mm)	68.3 ± 16.9	76.7 ± 16.4 *	56.2 ± 8.0	36.5 ± 15.7	1.51 ± 0.37	*Large* +
Body Composition						
Lean Mass (kg)	86.6 ± 9.5	92.3 ± 5.7 *	78.5 ± 7.9	17.6 ± 6.7	2.06 ± 0.40	*Very Large* +
Fat Mass (kg)	18.3 ± 5.0	21.1 ± 4.5 *	14.3 ± 1.8	48.2 ± 16.0	1.87 ± 0.39	*Large* +
Fat % (%)	16.6 ± 2.5	17.8 ± 2.4 *	14.8 ± 1.2	20.4 ± 8.2	1.51 ± 0.37	*Large* +
Bone Mass (kg)	3.9 ± 0.4	4.1 ± 0.4 *	3.6 ± 0.3	13.4 ± 6.0	1.45 ± 0.36	*Large* +
BMD (g/cm^2^)	1.37 ± 0.08	1.39 ± 0.09	1.34 ± 0.06	3.8 ± 3.4	0.67 ± 0.33	*Moderate* +

Mean ± SD reported for all players, forwards and backs. 95% CL = 95% confidence limit, Sum8SF = sum of eight site skinfolds, BMD = bone mineral density. * Significant difference (*p* ≤ 0.05) compared to backs. Note: + or – indicates that forwards demonstrated a higher or lower value than backs.

**Table 2:** Fitness characteristics of elite professional RU players.

	All Players (*n* = 39)	Position		% Diff (±95% CL)	Effect Size (±95% CL)	Qualitative Inference
		Forwards (*n* = 23)	Backs (*n* = 16)			
Strength						
Squat 1RM (kg)	189.3 ± 25.1	197.2 ± 26.5 *	178.1 ± 18.3	10.7 ± 8.4	0.81 ± 0.34	*Moderate* +
Bench Press 1RM (kg)	141.5 ± 14.6	146.1 ± 14.2 *	135.0 ± 12.8	8.2 ± 6.7	0.81 ± 0.34	*Moderate* +
Chin Up BW + AW (kg)	150.4 ± 13.1	155.0 ± 12.1 *	143.8 ± 12.0	7.8 ± 5.7	0.93 ± 0.34	*Moderate* +
Power						
CMJPP (W)	4889.3 ± 636.5	5151.8 ± 620.5 *	4512.1 ± 451.9	14.2 ± 8.1	1.15 ± 0.35	*Moderate* +
CMJRP (W/kg)	45.8 ± 7.2	44.7 ± 7.8	47.4 ± 6.3	−5.7 ± 9.3	−0.38 ± 0.33	*Small* −
CMJ40PP (W)	4568.7 ± 509.1	4756.5 ± 549.7 *	4298.7 ± 285.4	10.7 ± 6.4	0.99 ± 0.34	*Moderate* +
CMJ40RP (W/kg)	42.9 ± 6.5	41.3 ± 6.8	45.2 ± 5.3	−8.8 ± 8.2	−0.64 ± 0.33	*Moderate* −
Broad Jump (cm)	261.3 ± 18.6	252.3 ± 25.3 *	269.0 ± 13.0	−6.2 ± 4.5	−0.79 ± 0.34	*Moderate* −
Speed						
Sprint 10 m (s)	1.73 ± 0.09	1.78 ± 0.08 *	1.66 ± 0.04	7.5 ± 2.2	1.99 ± 0.40	*Large* +
Sprint 20 m (s)	3.02 ± 0.17	3.11 ± 0.15 *	2.88 ± 0.07	8.0 ± 2.5	1.90 ± 0.39	*Large* +
Aerobic Fitness						
Yo-Yo Distance (m)	1812.3 ± 454.3	1516.5 ± 291.9 *	2237.5 ± 266.3	−32.2 ± 6.8	−2.56 ± 0.44	*Very Large* −

Mean ± SD reported for all players, forwards and backs. 95% CL = 95% Confidence Limit, % Diff = % difference, 1RM = one-repetition maximum, BW+AW = bodyweight plus added weight, CMJPP = bodyweight countermovement jump peak power, CMJRP = bodyweight countermovement jump relative power, CMJ40PP = countermovement jump with 40kg peak power, CMJ40RP = countermovement jump with 40kg relative power. * Significant difference (*p* ≤ 0.05) compared to backs. Note: + or – indicates that forwards demonstrated a higher or lower value than backs.

**Table 3:** Relationships between physical and fitness characteristics within forwards (*n* = 23) and backs (*n* = 16).

	Squat 1RM	Bench Press 1RM	Chin Up 1RM	CMJ PP	CMJ RP	CMJ40 PP	CMJ40 RP	Broad Jump	10 m Sprint	20 m Sprint	Yo-Yo Distance
Forwards											
Demographics											
Age	0.04	0.08	0.20 ^*^	−0.33 ^§^	−0.21 ^*^	−0.33 ^§^	−0.21 ^*^	−0.48 ^§^	0.46 ^§^	0.46 ^§^	−0.23 ^*^
Experience	−0.09	0.05	0.16 ^*^	−0.48 ^§^	−0.30 ^§^	−0.49 ^§^	−0.31 ^§^	−0.51 ^‡^	0.47 ^§^	0.47 ^§^	−0.07
Anthropometrics											
Height	−0.22 ^*^	−0.07	0.23 ^*^	0.14 ^*^	0.02	0.16 ^*^	0.00	0.39 ^§^	−0.03	−0.10 ^*^	0.47 ^§^
Body Mass	0.59 ^‡^	0.27 ^*^	−0.05	−0.43 ^§^	−0.78 ^†^	−0.36 ^§^	−0.76 ^†^	−0.28 ^*^	0.69 ^‡^	0.69 ^‡^	−0.42 ^§^
Sum8SF	0.49 ^§^	0.16 ^*^	−0.16 ^*^	−0.69 ^‡^	−0.82 ^†^	−0.67 ^‡^	−0.84 ^†^	−0.62 ^‡^	0.75 ^†^	0.75 ^†^	−0.50 ^‡^
Body Comp											
Lean Mass	0.52 ^‡^	0.28 ^*^	0.07	−0.39 ^§^	−0.74 ^†^	−0.27 ^*^	−0.68 ^‡^	−0.24 ^*^	0.62 ^‡^	0.63 ^‡^	−0.30 ^§^
Fat Mass	0.69 ^‡^	0.23 ^*^	−0.19 ^*^	−0.46 ^§^	−0.73 ^†^	−0.45 ^§^	−0.75 ^†^	−0.44 ^§^	0.69 ^‡^	0.71 ^†^	−0.66 ^‡^
Fat %	0.68 ^‡^	0.20 ^*^	−0.24 ^*^	−0.45 ^§^	−0.66 ^‡^	−0.49 ^§^	−0.71 ^†^	−0.47 ^§^	0.65 ^‡^	0.67 ^‡^	−0.70 ^†^
Bone Mass	0.14 ^*^	0.19 ^*^	0.15 ^*^	−0.18 ^*^	−0.33 ^§^	−0.08	−0.27 ^*^	−0.00	0.13 ^*^	0.13 ^*^	−0.13 ^*^
BMD	0.17 ^*^	0.17 ^*^	0.18 ^*^	−0.16 ^*^	−0.27 ^*^	−0.09	−0.22 ^*^	−0.13 ^*^	0.12 ^*^	0.15 ^*^	−0.20 ^*^
Backs											
Demographics											
Age	0.19 ^*^	−0.27 ^*^	0.11 ^*^	−0.47 ^§^	−0.40 ^§^	−0.44 ^§^	−0.30 ^§^	−0.19 ^*^	0.33 ^§^	0.51 ^‡^	−0.09
Experience	−0.07	−0.41 ^§^	0.03	−0.46 ^§^	−0.30 ^§^	−0.39 ^§^	−0.16 ^*^	−0.17 ^*^	0.24 ^*^	0.40 ^§^	0.05
Anthropometrics											
Height	0.18 ^*^	0.02	0.64 ^‡^	−0.00	−0.56 ^‡^	0.15 ^*^	−0.56 ^‡^	0.41 ^§^	−0.10 ^*^	−0.21 ^*^	0.23 ^*^
Body Mass	0.56 ^‡^	0.42 ^§^	0.88 ^†^	0.07	−0.70 ^†^	0.03	−0.85 ^†^	0.00	0.03	−0.08	−0.06
Sum8SF	0.03	0.25 ^*^	−0.09	0.16 ^*^	0.17 ^*^	0.02	0.07	−0.45 ^§^	0.03	0.00	−0.54 ^‡^
Body Comp											
Lean Mass	0.60 ^‡^	0.47 ^§^	0.89 ^†^	0.13 ^*^	−0.65 ^‡^	0.07	−0.82 ^†^	0.05	−0.00	−0.12 ^*^	−0.05
Fat Mass	0.21 ^*^	0.28 ^*^	0.58 ^‡^	−0.05	−0.61 ^‡^	−0.02	−0.68 ^‡^	−0.32 ^§^	0.29 ^*^	0.14 ^*^	−0.13 ^*^
Fat %	−0.32 ^§^	−0.12 ^*^	−0.17 ^*^	−0.19 ^*^	−0.13 ^*^	−0.12 ^*^	−0.06	−0.48 ^§^	0.40 ^§^	0.30 ^§^	−0.20 ^*^
Bone Mass	0.34 ^§^	0.32 ^§^	0.63 ^‡^	0.21 ^*^	−0.33 ^§^	0.26 ^*^	−0.42 ^§^	0.19 ^*^	0.01	−0.02	0.29 ^*^
BMD	0.08	0.29 ^*^	0.23 ^*^	0.49 ^§^	0.26 ^*^	0.50 ^‡^	0.16 ^*^	0.18 ^*^	−0.15 ^*^	−0.13 ^*^	0.34 ^§^

1RM = one repetition maximum, CMJPP = bodyweight countermovement jump peak power, CMJRP = bodyweight countermovement jump relative power, CMJ40PP = countermovement jump with 40 kg peak power, CMJ40RP = countermovement jump with 40 kg relative power, Sum8SF = sum of eight site skinfolds, Body Comp = body composition, BMD = bone mineral density. Magnitudes of correlations: ^†^ *very large* (0.70–0.89), ^‡^ *large* (0.50–0.69), ^§^ *moderate* (0.30–0.49), * *small* (0.10–0.29), all other results are *trivial* (< 0.09).
